# Superior 12-month progression-free survival with frontline DVd versus VRd in patients with newly diagnosed multiple myeloma: a multicenter propensity score-matched analysis

**DOI:** 10.1186/s12967-026-08637-6

**Published:** 2026-08-12

**Authors:** Huinan Jiang, Qian Li, Liru Wang, Xuelin Dou, Qing Leng, Miao Miao, Ying Yang, Ming Zhang, Yingchun Li, Jia Li, Rong Zhang, Ying Lv, Zhuogang Liu, Huihan Wang, Yang Li, Rong Li, Rui Zhang, Fei Tian, Yu Jiang, Jingjun Wang, Min Wang, Xiaohong Wang, Xianming He, Jin Lu, Aijun Liao

**Affiliations:** 1 Department of Hematology, Shengjing Hospital of China Medical University, Shenyang, China; 2 Peking University Institute of Hematology, Peking University People’s Hospital, Beijing, China; 3 Department of Hematology, Fu Xing Hospital of Capital Medical University, Beijing, China; 4 Department of Hematology, Anshan Central Hospital, Anshan, China; 5 Department of Hematology, Panjin Liaoyou Gemstone Flower Hospital, Panjing, China; 6 Department of Hematology, The First Affiliated Hospital of China Medical University, Shenyang, China; 7 Department of Hematology, Huludao Central Hospital, Huludao, China; 8 Department of Hematology, Panjin Central Hospital, Panjing, China; 9 Department of Hematology, Tieling Central Hospital, Tieling, China; 10 Department of Hematology, Liaoning Health Industry Group Bengang General Hospital, Benxi, China; 11 Department of Hematology, Chaoyang Central Hospital, Chaoyang, China

## Abstract

**Background:**

Multiple myeloma, a malignant plasma cell disorder, has undergone remarkable therapeutic advancement. The lenalidomide-bortezomib-dexamethasone (VRd) regimen has been firmly established as one of the standard induction therapies for newly diagnosed multiple myeloma (NDMM) patients, owing to its substantial clinical benefits. Despite its superiority in achieving good responses, VRd harbors intrinsic limitations in high relapse risk, and treatment-related toxicities. Dara (Daratumumab)-containing regimens are prioritized as frontline treatment options per international guidelines. While the quadruplet Dara-VRd combination further improves clinical efficacy, it carries notable safety and tolerability burdens and is suboptimal for vulnerable patient subgroups: it is poorly tolerated by elderly or frail individuals, requires complicated dose modifications for patients with renal impairment, and impairs hematopoietic stem cell mobilization in transplant-eligible candidates. The triplet daratumumab-bortezomib-dexamethasone (DVd) regimen has yielded encouraging anti-tumor activity in patients with relapsed/refractory multiple myeloma (RRMM), which motivated us to explore its clinical performance in the frontline setting. The present study aimed to compare the efficacy and safety of frontline DVd versus VRd induction, to generate real-world clinical evidence guiding individualized treatment decision-making for NDMM.

**Objective:**

To compare the efficacy, safety and real-world applicability of DVd versus VRd in NDMM patients.

**Methods:**

This multicenter prospective study enrolled 206 NDMM patients (DVd: *n* = 98; VRd: *n* = 108) from 12 Chinese centers (March 2023–March 2025). Propensity score matching (PSM; covariates: age, serum creatinine, Durie-Salmon stage, ISS stage) generated a balanced cohort (70 patients/group). Key endpoints: overall response rate (ORR), renal response rate (RRR), time to renal recovery (TTRR), progression-free survival (PFS), duration of response (DOR), and adverse events (AEs; NCI CTCAE v5.0).

**Results:**

Both groups had high completion rates after induction (95.9% [94/98] vs. 94.4% [102/108], *p* = 0.623). **ORR** was comparable in the original cohort (DVd 97.9% vs. VRd 94.1%, *p* = 0.282), but DVd achieved faster response (median time to response: 1.85 vs. 2.27 months, *p* = 0.002), despite worse baseline characteristics. In the propensity score-matched cohort, the two groups also had similar ORR (DVd 97.1% vs. VRd 97.0%, *p* = 1.00) with comparable deep remission rates (MRD negativity: 4.3% vs. 7.3%; sCR/CR: 30.4% vs. 32.3%). **Renal response:** Despite lower mean baseline eGFR in DVd group (23.7 vs. 32.7 mL/min, p < 0.001), magnitude of eGFR improvement was comparable (ΔeGFR: 15.2 vs. 12.7 mL/min, p = 0.48) in the original cohort. In the propensity score-matched cohort, 78.6% (22/28) in DVd and 95.0% (19/20) in VRd achieved response (renal-CR: 50.0% vs. 70.0%; renal-PR: 7.1% vs. 15.0%; renal-MR: 21.4% vs. 10.0%, p = 0.207). **PFS:** In the original cohort, the 12-month PFS rate was 92.8% in the DVd group and 79% in the VRd group. In the propensity score-matched cohort, the 12-month PFS rate in DVd and VRd group was 91.9% vs. 84%. Subgroup analyses confirmed PFS benefits in patients with high-risk genetics, ISS stage III, and transplant-eligible patients. **DOR:** In the original cohort, the 12-month DOR rate was 92.5% in the DVd group and 74.6% in the VRd group. In the propensity score-matched cohort cohort, the 12-month DOR rate in DVd and VRd group was 91.9% vs. 84%. Subgroup analyses confirmed DOR benefits in patients with high cytogenetic risk, ISS stage III, and transplant-eligible patients. **Safety:** Across both the original and PSM cohorts, the DVd regimen had relatively fewer severe adverse reactions than the VRd regimen with no statistical significance. After PSM, the patients in DVd group vs. VRd group: grade 3–4 neutropenia (5.71% vs. 10%, p = 0.346) and thrombocytopenia (10% vs. 14.29%, p = 0.438), grade 3–4 ALT elevation (7.14% vs. 10%, p = 0.546).

**Conclusion:**

DVd demonstrates faster response, superior PFS and DOR, accompanying with relatively less toxicity in NDMM patients. These real-world data support DVd as a frontline option for NDMM patients.

**Supplementary Information:**

The online version contains supplementary material available at 10.1186/s12967-026-08637-6.

## Introduction

Multiple myeloma (MM), the second most common hematologic malignancy, arises from clonal plasma cell proliferation in bone marrow, leading to osteolytic lesions, renal dysfunction, anemia, and hypercalcemia [1]. Globally, MM accounts for approximately 14% of hematologic cancers [2], with an annual incidence of 2.1 cases per 100,000 individuals [3] and a median age at diagnosis of 70 years. Over the past three decades, the MM burden in China has steadily increased [4]. The therapeutic landscape for NDMM has shifted from traditional chemotherapy to novel agent-based regimens which revolutionized patient outcomes. Despite therapeutic advancements, MM remains an incurable cancer characterized by high relapse rates driven by clonal evolution and microenvironment escape-mediated drug resistance [5]. 

Regimens constituted by proteasome inhibitors (PIs) and/or immunomodulatory drugs (IMiDs) including bortezomib and lenalidomide, have ushered in a transformative era for MM management. The combination of lenalidomide, bortezomib, and dexamethasone (VRd) has been established as a cornerstone of frontline therapy, with robust clinical data supporting its efficacy in both transplant-eligible and ineligible patients. Clinical trials have demonstrated that VRd achieves high rates of overall response rate (ORR) of 82–90%, alongside a 48-month progression-free survival (PFS) rate of 67.7%, which significantly outperforms conventional chemotherapy [6, 7]. Nevertheless, multiple unmet clinical gaps persist under this treatment paradigm. A substantial proportion of patients failed to achieve deep and durable responses, only few patients have the chance to obtain minimal residual disease (MRD) negativity [8], and renal recovery rates in patients with myeloma-related kidney injury remain suboptimal. Non-targeted therapies could cause significant toxicities, including peripheral neuropathy caused by PIs and IMiDs, myelosuppression, fatigue, neuropathy, and thromboembolic events related to IMiDs like lenalidomide [9, 10]. Furthermore, VRd demonstrates insufficient anti-myeloma activity in patients harboring high-risk cytogenetic abnormalities, particularly per the updated 2024 IMS/IMWG risk stratification criteria. These persistent clinical limitations underscore an urgent need to develop more effective, better-tolerated frontline therapeutic combinations [11]. 

Daratumumab, an anti-CD38 monoclonal antibody, disrupts MM cell via multiple mechanisms: complement-dependent cytotoxicity (CDC), antibody-dependent cellular cytotoxicity (ADCC), Antibody-Dependent Cellular Phagocytosis (ADCP) and inhibition of CD38-mediated nicotinamide adenine dinucleotide (NAD+) metabolism [12]. At present, daratumumab-containing intensified regimens have delivered landmark efficacy outcomes in pivotal phase 3 trials and are endorsed as core frontline treatment backbones by the NCCN and EHA-EMN clinical guidelines. The MAIA trial validated prominent survival improvements with the DRd regimen for transplant-ineligible NDMM patients [13], whereas the PERSEUS and CASSIOPEIA trials confirmed deeper remission and superior efficacy of D-VRd and D-VTd versus control arms, respectively, in transplant-eligible populations [14, 15]. Despite the encouraging results in clinical trials, broad real-world adoption of daratumumab-based quadruplet regimens is hampered by several critical limitations. First, the patients enrolled in clinical trials were well selected, which might not represent the true complex characteristic of real-world NDMM patients. Second, most of the patients included in these clinical trials were from the US or the European countries whose baseline characteristics may be different from East Asian or Chinese patient cohorts. Third, lenalidomide — a core component of DRd and D-VRd — poses substantial restrictions for patients complicated by renal impairment, a complication observed in 20%–40% of Chinese NDMM patients [16]. Individuals with severe renal dysfunction or those receiving dialysis require drastic lenalidomide dose reduction or are even contraindicated from receiving the agent, thereby blunting its full therapeutic potential. Beyond these constraints, quadruplet combinations such as D-VRd, D-VTd and D-KRd, though capable of improving response depth and PFS, are linked to markedly elevated treatment-related toxicities, including higher rates of grade III/IV neutropenia and greater risks of opportunistic infections, which compromise long-term treatment tolerability and patients’ quality of life. According to data published earlier, more than 60% of NDMM patients in China were frail and could not tolerate quadruplet regimens [17]. Notably, the CASTOR trial established that adding daratumumab to the Vd backbone delivers robust clinical benefits for patients with relapsed/refractory MM [18], and subsequent real-world analyses have validated the DVd triplet as an effective regimen with a favorable safety profile in relapsed disease settings. Preclinical research indicates that bortezomib undergoes non-renal metabolic clearance, conferring unique advantages for patients with renal impairment — a finding further corroborated by clinical and post-marketing observational data. Collectively, these observations motivate the development of a lenalidomide-free frontline regimen that balances potent anti-tumor efficacy and reduced toxicity. The present study therefore aimed to systematically compare the efficacy and safety of frontline DVd against the standard VRd induction regimen, with the goal of generating high-quality real-world evidence to guide individualized clinical decision-making for Chinese NDMM patients.

## Methods

### Study design and ethics

This multicenter prospective observational cohort study obtained primary ethical approval from the Ethics Committee of Shengjing Hospital of China Medical University (coordinating center) in 2023 (No. 2023PS090J) and completed domestic research filing that year. All patient enrollment, treatment and follow-up between March 2023 and March 2025 were conducted under this original ethical authorization. To meet international publication and registry standards, we acquired supplementary research ethical approval in early 2025 (No. 2025PS795K) and completed registration in the Chinese Clinical Trial Registry (ChiCTR2500102869) on May 21, 2025. We fully disclosed the March 2023 study start date and uploaded the full 2023 ethics documentation during registry submission, which was reviewed and approved by the registry office. All participants provided written informed consent before any study procedures, in accordance with the Declaration of Helsinki. A timeline of the two-stage ethical and registration process is provided in Supplementary Fig. S2.

### Patient eligibility

Treatment-naive adult patients with newly diagnosed multiple myeloma (NDMM) were prospectively enrolled across 12 Northern Chinese centers from March 2023 to March 2025. A full list of participating centers is available in Supplementary Materials. Inclusion and exclusion criteria are shown below. Inclusion criteria: [1] Age ≥ 18 years; [2] NDMM diagnosed per the 2022 Chinese Multiple Myeloma Guidelines with measurable disease: serum M-protein ≥ 1 g/dL, 24-h urinary M-protein ≥ 200 mg, or abnormal free light chain ratio with involved free light chain ≥ 10 mg/dL; [3] Adequate baseline organ function: total bilirubin ≤ 1.5× ULN, AST/ALT ≤ 2× ULN, CK-MB/troponin < 2× ULN. Exclusion criteria: [1] Active HBV (HBsAg positive, HBV DNA ≥ 2000 IU/mL) or positive HCV RNA; [2] Baseline grade ≥ 2 peripheral neuropathy; [3] Contraindications to study regimens; [4] Pregnancy or breastfeeding; [5] COPD/asthma with FEV1 < 50% predicted.

### Treatment regimens

Patients were assigned to DVd or VRd based on physicians’ decision and patients’ choice:

- DVd: Dara 16 mg/kg IV (weekly, cycles 1–2; every 2 weeks, cycles 3–4) + bortezomib 1.3 mg/m² SC (days 1,8,15,22) + dexamethasone 20–40 mg IV/PO (days 1–2,8–9,15–16,22–23; 20 mg for age ≥ 75 years or BMI < 18).
- VRd: Lenalidomide 25 mg PO (days 1–21; adjusted for eGFR: 10–15 mg if eGFR 30–60 mL/min, 5 mg if eGFR < 30 mL/min) + same bortezomib/dexamethasone as DVd arm.

Cycles were 28 days; treatment of DVd or VRd continued for 4 cycles. (Table 1)

**Table 1:** Trial design

Phase	Treatment
Cycle 1–4: DVd regimen or VRd regimen	Dara(16 mg/kg IV) cycle 1–2, once a week cycle 3–4, twice a week Lenalidomide (25 mg-adjusted according to renal function PO) d1-21 Bortezomib (1.3 mg/ m^2^ SC) d1, 8, 15, 22 Dexamethasone (20 mg IV/PO) d1-2, 8–9, 15–16, 22–23

### Efficacy and safety assessments

The efficacy evaluation of the intent-to-treat (ITT) population was conducted in accordance with the International Myeloma Working Group (IMWG) criteria [19, 20]. Response assessments were performed at the start of each cycle induction cycle. The MRD status was evaluated by multiparameter flow cytometry (MFC) with a sensitivity of 10^− 5^ after the patient achieves strict complete response (sCR). Renal impairment (RI) was defined as serum creatinine ≥ 2 mg/dL (≥ 177 µmol/L) or eGFR < 60 mL/min/1.73 m² (CKD-EPI/MDRD formulas). Renal response rate (RRR) was judged by IMWG standards [21]. Time to renal recovery (TTRR) was the interval from treatment onset to first sustained renal response; non-responders and lost-to-follow-up patients were censored at their last renal assessment. PFS was calculated from treatment initiation to relapse, progression or death [22]. Duration of response (DOR) covered the first objective response (CR/VGPR/PR) until progression or all-cause death. Patients without events at cutoff were censored at their last response review [23]. Adverse events were graded by NCI CTCAE V5.0, with focus on peripheral neuropathy, infection and hematologic toxicity. All VRd patients received aspirin/warfarin for thromboprophylaxis, and all subjects took acyclovir to prevent herpes zoster.

### Statistical analysis

Propensity score matching balanced baseline covariates (age, serum creatinine, Durie-Salmon stage, ISS stage) between cohorts. Categorical variables were compared with χ²/Fisher’s exact tests; continuous variables with t-tests/Mann-Whitney U tests. PFS and DOR was estimated via Kaplan-Meier method and compared with log-rank test. Multivariate logistic regression assessed interactions between baseline factors and treatment efficacy. All analyses used SPSS 27.0; two-sided *p* < 0.05 was significant.

## Results

### Comparison between DVd and VRd cohorts

**Fig. 1:**
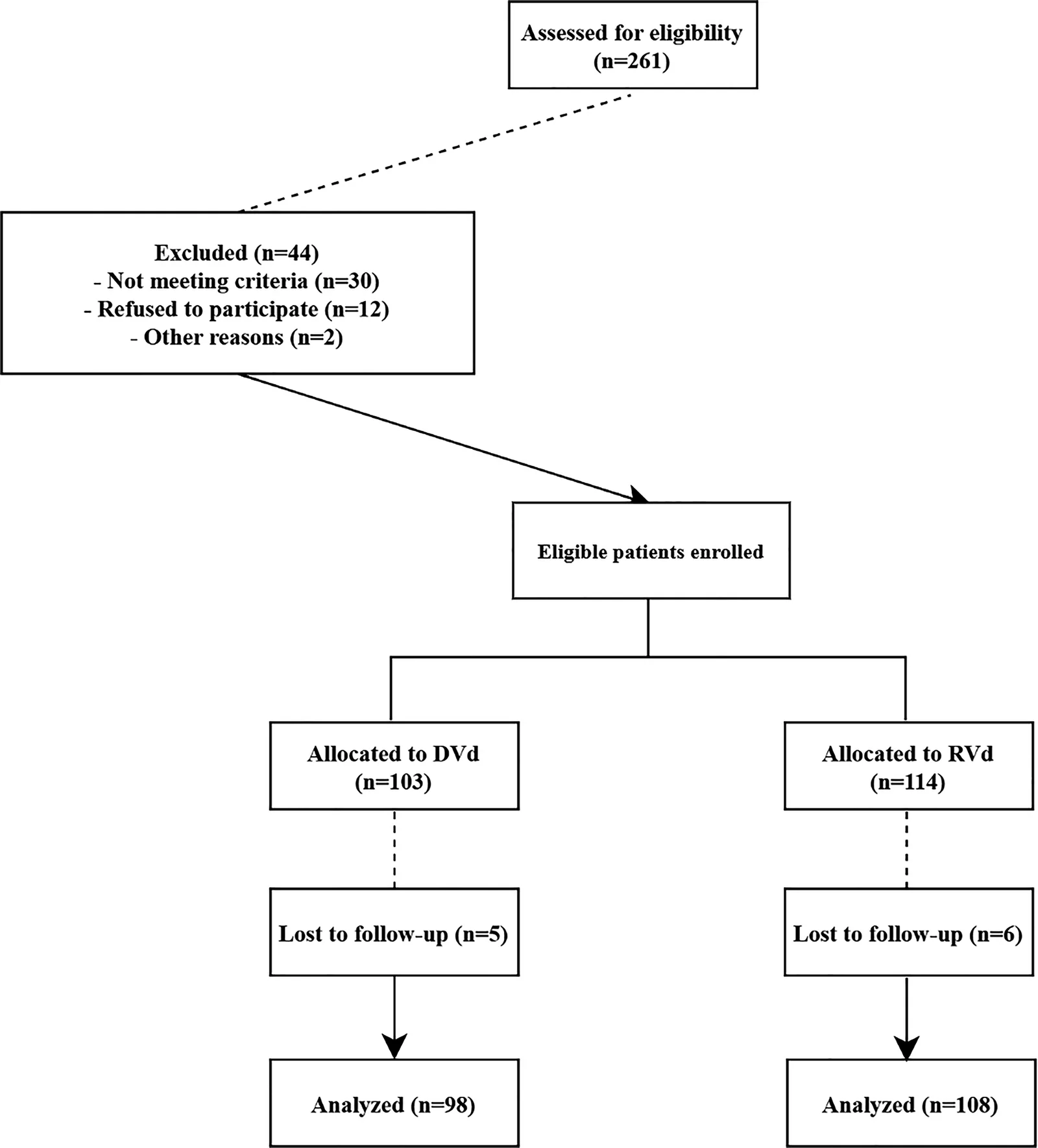
A CONSORT flowchart detailing the participant flow from screening to final analysis

Between March 2023 and March 2025, 206 eligible patients formed the original cohort and received either DVd or VRd induction (Fig. 1). In the original cohort, the DVd group had worse baseline characteristics compared with VRd arm: older median age (64 vs. 61 years, *p* = 0.034), higher proportion of ISS II/III (85.7% vs. 77.7%, *p* = 0.033), higher proportion of severe renal impairment (eGFR < 60 mL/min: 55.9% vs. 25.3%, *p* < 0.001), and more patients with high-risk cytogenetics (defined as del(17p), t(4;14), t(14;16), t(14;20): 50.0% vs. 33.3%, *p* = 0.105; Table 2A). Baseline transplant eligibility differed markedly: 53 eligible / 45 ineligible patients in the DVd group versus 99 eligible / 9 ineligible patients in the VRd group. Post-induction HSCT was administered to 9 DVd patients (3 of whom became transplant-eligible after treatment) and 27 VRd patients. After 1:1 propensity score matching (70 patients per arm), all baseline covariates were well balanced (*p* > 0.05; Table 2B). In the matched cohort, 42 DVd and 62 VRd patients were transplant-eligible; 6 DVd and 10 VRd patients received subsequent HSCT.

**Table 2A:** Baseline patient characteristics of the original cohort

Characteristics	DVd（N=98)	VRd(N=108)	P value
Median age-yr (IQR)	64 (35-83)	61 (37-80)	0.034
Age category-n (%)			
≤65 yr	56 (57.1)	66 (61.1)	
>65 yr	42 (42.9)	42 (38.9)	
Sex-n(%)			
Male	37 (37.8)	60（55.6）	
Female	61 (62.2)	48（44.4）	
MM subtyoe-n/total n (%)			
IgG	48/98（49.0）	59/108 (64.5)	
IgA	19/98（19.4）	28/108（25.9）	
IgD	4/98（4.1）	4/108（3.8）	
IgM	1/98 (1.0)	0/108 (0)	
Light chain	24/98 (24.5)	15/108 (13.9)	
Non-secretory	2/98 (2.0)	2/108 (1.9)	
Durie-Salmon Staging System stage-n/total n (%)			0.131
I	8/98 (8.1)	3/108 (2.8)	
II	7/98 (7.1)	13/108 (12)	
III	83/98 (84.8)	92/108 (85.2)	
International Staging System stage-n/total n (%)			0.033
I	12/96 (12.2)	21/105 (19.4)	
II	19/96 (19.4)	32/105 (29.6)	
III	65/96 (66.3)	52/105 (48.1)	
Missing	2 (2.1)	3 (2.9)	
High LDH (>250U/L)-n(%)	15 (16.1)	12 (12.9)	0.446
Cytogenetic risk-n/total n(%)			0.105
High-risk	49 (50.0)	36 (33.3)	
Standard-risk	37 (37.8)	45 (41.7)	
Missing	12 (12.2)	27 (25.0)	
del(17p)-n/total n(%)	20 (20.4)	10 (9.3)	
t(4;14)-n/total n(%)	17 (17.3)	10 (9.3)	
t(14;16)-n/total n(%)	10 (10.2)	6 (5.6)	
t(14;20)-n/total n(%)	5 (5.1)	5 (4.6)	
High copy number amp 1q-n/total n(%)	6 (6.1)	9 (8.3)	
Extramedullary disease-n(%)	11 (11.2)	14 (13.0)	0.833
Creatinine clearance-n(%)			<0.001
＜60 mL/min	56 (55.9)	25 (23.2)	
≥60 mL/min	42 (44.1)	75 (69.4)	
Missing	0	8 (7.4)	

**Table 2B:** Baseline patient characteristics of propensity score-matched cohort

Characteristics	DVd（N=70)	VRd(N=70)	P value
Median age-yr (IQR)	64 (35-83)	64 (46-80)	0.109
Age category-n (%)			
≤65 yr	39 (55.7)	33 (47.1)	
>65 yr	31 (44.3)	37 (52.9)	
Sex-n(%)			
Male	27 (38.6)	40（57.1）	
Female	43 (61.4)	30（42.9）	
MM subtyoe-n/total n (%)			
IgG	38/70（54.3）	38/70（54.3）	
IgA	14/70（20.0）	20/70（28.6）	
IgD	2/70（2.85）	3/70（4.2）	
IgM	1/70 (1.4)	0/70 (0)	
Light chain	13/70 (18.6)	9/70 (12.9)	
Non-secretory	2/70 (2.85)	0/70 (0)	
Durie-Salmon Staging System stage-n/total n (%)	0.07		
I	7/70 (10.0)	3/70（4.3）	
II	6/70 (8.6)	7/70（10.0）	
III	57/70（81.4)	60/70（85.7）	
International Staging System stage-n/total n (%)	0.92		
I	12/70 (17.1)	11/70 15.7)	
II	17/70 (24.3)	19/70 (27.1)	
III	41/70 (58.6)	40/70 (57,2)	
High LDH (>250U/L)-n(%)	9 (15.4)	11 (17.7)	0.253
Cytogenetic risk-n/total n(%)	0.516		
High-risk	32/70 (45.7)	24/70 (34.3)	
Standard-risk	27/70 (38.6)	26/70 (37.1)	
Missing	11/70 (15.7)	20/70 (28.6)	
del(17p)-n/total n(%)	15/70 (21.4)	8/70 (11.4)	
t(4;14)-n/total n(%)	11/70 (15.7)	6/70 (8.6)	
t(14;16)-n/total n(%)	9/70 (12.9)	5/70 (7.1)	
t(14;20)-n/total n(%)	5/70 (7.1)	5/70 (7.1)	
High copy number amp 1q-n/total n(%)	21/70 (30.0)	26/70 (37.1)	
Extramedullary disease-n(%)	8/70 (11.4)	5/70 (7.1)	0.367
Creatinine clearance-n(%)	0.324		
＜60 mL/min	29 (41.4)	23 (32.9)	
≥60 mL/min	41 (58.6)	46 (65.7)	
Missing	0	1 (1.4)	

### Treatment completion

Four-cycle completion rates were high in both cohorts: 95.9% (94/98) DVd vs. 94.4% (102/108) in the original cohort (*p* = 0.623), and 98.6% (69/70) DVd vs. 97.1% (68/70) in the matched cohort, demonstrating good real-world tolerability of bortezomib-based triplets. Primary discontinuation cause was inadequate response. Four DVd patients stopped early: one switched therapy for stable disease (SD) at cycle 3; one relapsed at cycle 3 despite initial very good partial response (VGPR) with pleural infiltration/effusion; one transferred for autologous HSCT without cycle-3 assessment; one progressed to progressive disease (PD) at cycle 3 and changed regimens. Six VRd patients discontinued early: four had no response after three cycles, one developed PD at cycle 3, and one was lost to follow-up.

### Efficacy

#### Overall remission and MRD negativity

ORR, ≥CR and ≥VGPR were similar between arms. Original-cohort ORR was 97.9% (DVd) vs. 94.1% (VRd) (OR = 2.875, *p* = 0.282), with numerically faster response onset in DVd vs. RVd (median 1.85 vs. 2.27 months, *p* = 0.002), beneficial for organ-impaired patients. Ordinal multinomial logistic regression (proportional odds model) was used to analyze the association between the two regimens and 4-level ordinal efficacy grades (MRD negative rate, CR rate, VGPR rate and PR rate). The parallel lines test (χ²=1.598, df = 3, *p* = 0.66) confirmed the model’s appropriateness by not violating the parallel lines assumption. The results (Estimate=-0.206, *p* = 0.432) indicated no statistically significant difference in efficacy grades between the two regimens, suggesting they yield similar efficacy outcomes (Table 3). DVd patients had worse baseline risks: older age (mean age: 63.90 ± 9.37 vs. 61.19 ± 8.92, *p* = 0.034), higher rates of concomitant renal impairment (57.14% vs. 25.25%, *p* < 0.001), and higher rates of high-risk cytogenetics (HRCA: 56.98% vs. 44.44%, *p* = 0.105), especially del(17p), yet still achieved robust deep remission.

In the propensity score-matched cohort, DVd retained shorter median time to response without statistical significance (1.98 months vs. 2.26 months, *p* = 0.108). No significant difference was found between DVd and VRd in terms of ORR (OR = 1.015, *p* = 1.00). Ordinal multinomial logistic regression (proportional odds model) was used to analyze the association between the two regimens and 4-level ordinal efficacy grades, too. The results (Estimate=-0.004, *p* = 0.991) once again confirmed that there was no difference in efficacy grading between the two regimens (Table 3). Two DVd patients achieved only SD: one with del(17p) switched to KPd (carfilzomib, pomalidomide, dexamethasone) and attained PR; another with multiple high-risk aberrations gained transient PR then PD, complicated by grade ≥ 3 infections and treatment interruptions. Two VRd SD patients were noted: one with poor adherence failed SPd (selinexor, pomalidomide, dexamethasone) salvage; the other transferred to another hospital. Overall, DVd exerted rapid, potent anti-tumor effects, enabling quick symptom control for patients with high tumor burden.

**Table 3:** Summary of best response achieved in DVd and VRd cohorts

Best overall response^a^, *N* (%)	Original cohort		Propensity score-matched cohort	
	DVd(*N* = 94)	VRd(*N* = 102)	DVd(*N* = 69)	VRd(*N* = 68)
MRD negativity	5 (5.3)	5 (4.9)	3 (4.3)	5 (7.3)
CR or better	30 (31.9)	31 (30.4)	21 (30.4)	22 (32.3)
VGPR	40 (42.6)	39 (38.2)	30 (43.5)	21 (30.9)
PR	17 (18.1)	21 (20.6)	13 (18.8)	18 (26.5)
ORR^b^	92 (97.9)	96 (94.1)	67 (97.1)	66 (97.0)
SD and PD	2 (2.1)	6 (5.9)	2 (2.9)	2 (3.0)

^a^Best response assessment by physician according to international Myeloma working group criteria. ^b^ORR include ≥ PR

#### Renal response

Renal response in the original cohort, was 79.2% (39/53) in DVd group and 95.2% (20/21) in VRd group (renal-CR: 37.7% vs. 71.4%; renal-PR: 15.1% vs. 14.3%; renal-MR: 26.4% vs. 9.5%). However, baseline renal function was significantly different between groups. In the DVd group, the mean baseline eGFR of patients with renal impairment was 23.7 ml/min/1.73 m², with 72% having severe renal impairment (eGFR<30 ml/min/1.73m^2^) and 43% having renal failure (eGFR<15 ml/min/1.73m^2^). In the VRd group, the mean baseline eGFR was 32.7 ml/min/1.73 m², with 52% having severe renal impairment and only 19% having renal failure. Four-cycle eGFR increments were similar (15.2 vs. 12.7 mL/min/1.73 m², *p* = 0.48). Matched cohort renal response: DVd 78.6% (22/28), VRd 95.0% (19/20). VRd trended better numerically, with no significant difference (*p* = 0.207, Table  4).

**Table 4:** Summary of RRR achieved in DVd and VRd cohorts

Renal response, *N* (%)	Original cohort		Propensity score-matched cohort	
	DVd(*N* = 53)	VRd(*N* = 21)	DVd(*N* = 28)	VRd(*N* = 20)
renal-CR	20 (37.7)	15 (71.4)	14 (50.0)	14 (70.0)
reanl-PR	8 (15.1)	3 (14.3)	2 (7.1)	3 (15.0)
renal-MR	14 (26.4)	2 (9.5)	6 (21.4)	2 (10.0)
overall renal response	42 (79.2)	20 (95.2)	22 (78.6)	19 (95.0)
no response	11 (20.8)	1 (4.8)	6 (21.4)	1 (5.0)

Stratified analysis by baseline eGFR (30–60, 15–30, < 15 mL/min/1.73 m²) revealed no intergroup disparities, constrained by small subgroup sizes (Table S1). Kaplan–Meier TTRR analysis yielded identical median 1.0‑month recovery across groups. VRd showed numerically shorter mean TTRR in the original cohort (1.24 vs. 1.85 months; HR = 0.60, 95%CI 0.35–1.02, *p* = 0.052, Fig. S1A) and matched cohort (1.25 vs. 1.82 months; HR = 0.63, 95%CI 0.34–1.17, *p* = 0.13, Fig. S1B), without statistical significance.

#### Progression-free survival

**Fig. 2:**
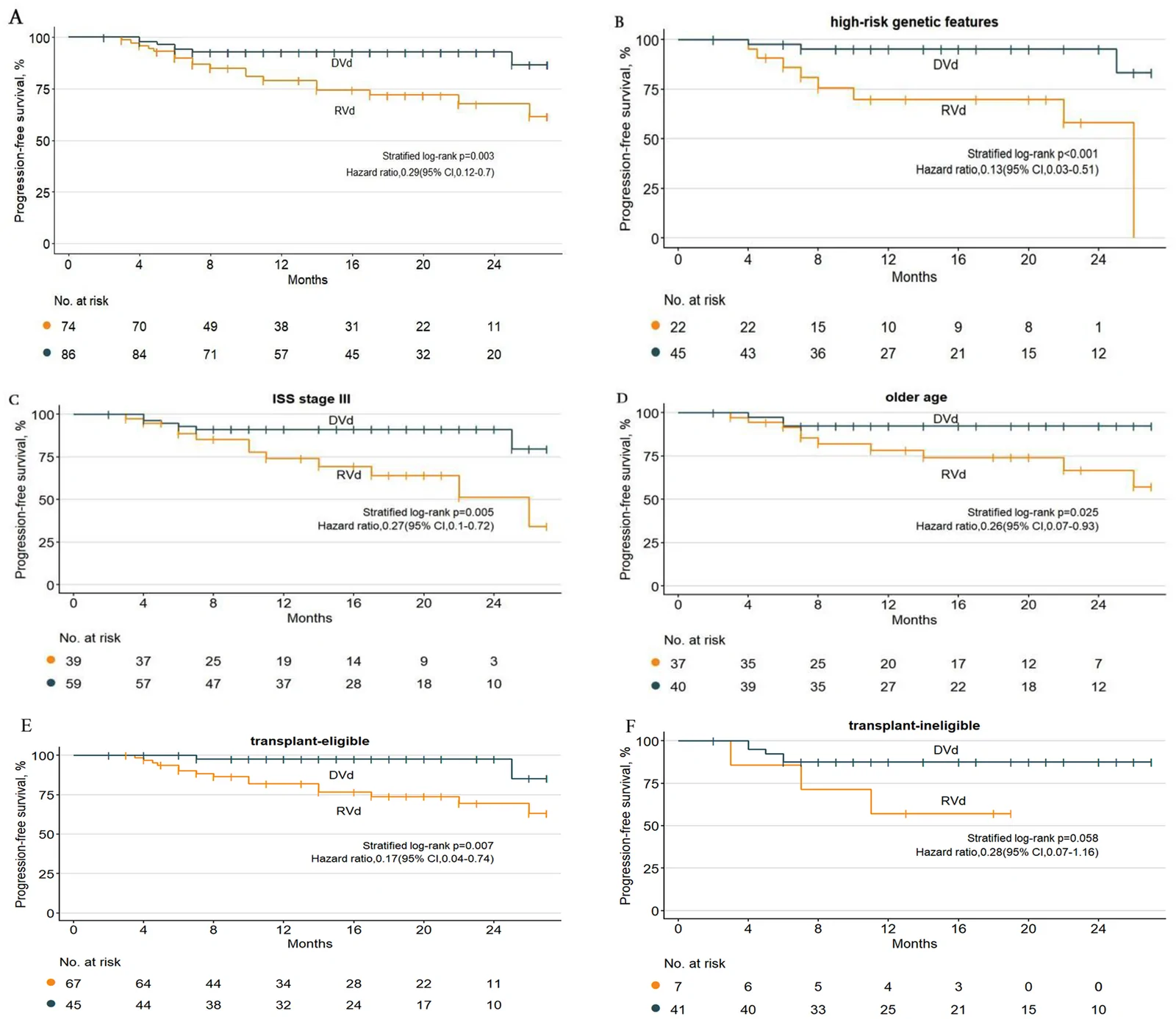
Panels **A**–**D** displayed Kaplan-Meier survival functions comparing the DVd and VRd regimens in the original cohort NDMM patients across distinct subgroups. **A**: The overall cohort of NDMM patients. **B**: NDMM patients with high-risk genetic features. **C**: NDMM patients with ISS stage III disease. **D**: NDMM patients with older age. **E**: TE-NEMM patients. **F**: TIE-NDMM patients. *Limitation: By June 2025, the DVd group had an average induction therapy duration of 8 months: high-risk genetic patients received Dara+lenalidomide or bortezomib+lenalidomide for maintenance, while standard-risk patients received lenalidomide monotherapy. The VRd group also had a 8-month average induction duration: high-risk patients were maintained with bortezomib+lenalidomide, and standard-risk patients with lenalidomide monotherapy. After achieving a therapeutic effect of PR or higher with induction therapy, 9 patients in the DVd group and 29 patients in the VRd group underwent autologous hematopoietic stem cell transplantation. The transplant-eligible and transplant-ineligible cohorts were stratified according to formal HSCT eligibility criteria, and this subgroup analysis was only provided as supplementary information

At the June 2025 cutoff, median progression-free survival (PFS) was unreached for both arms, with over half of patients free of progression. Kaplan–Meier analyses (transplant recipients excluded) were conducted for the full cohort and subgroups. Median follow-up was 17 months; 12-month PFS was 92.8% (DVd) vs. 79% (VRd). DVd reduced progression risk by 71% overall (HR = 0.29, 95%CI: 0.12–0.7, *p* = 0.003) (Fig. 2A), with consistent significant benefits in high-risk subgroups: high-risk cytogenetics (87% risk reduction, HR = 0.13, 95%CI: 0.03–0.51, *p* < 0.001, Fig. 2B), ISS III (HR = 0.27, 73% reduction, 95% CI: 0.1–0.72, *p* = 0.005, Fig. 2C), and age > 65 years (HR = 0.26, 74% reduction, 95% CI: 0.07–0.93, *p* = 0.025, Fig. 2D). DVd significantly improved PFS in transplant-eligible NDMM (TE-NDMM) (HR = 0.29, 71% risk reduction, 95%CI: 0.12–0.7, *p* = 0.007, Fig. 2E). For transplant-ineligible NDMM (TIE-NDMM) patients, DVd showed a favorable non-significant trend (HR = 0.28, 95%CI 0.07–1.16, *p* = 0.058, Fig. 2F). DVd offers robust PFS benefits for transplant-eligible patients and promising activity for transplant-ineligible patients.

**Fig. 3:**
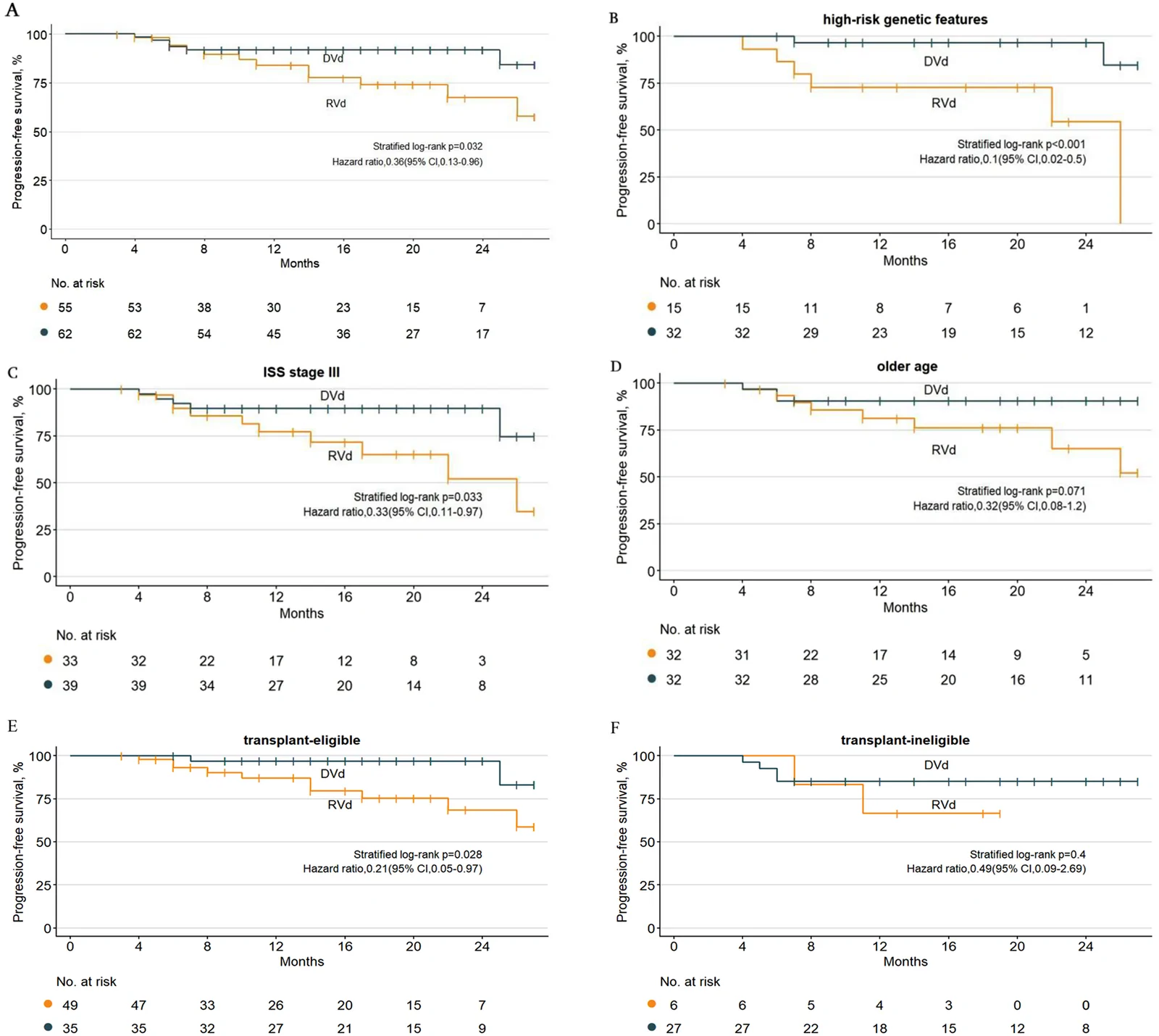
Panels **A**–**D** displayed Kaplan-Meier survival functions comparing the DVd and VRd regimens in the propensity score-matched cohort NDMM patients across distinct subgroups. **A**: The overall cohort of NDMM patients. **B**: NDMM patients with high-risk genetic features. **C**: NDMM patients with ISS stage III disease. **D**: NDMM patients with older age. **E**: TE-NEMM patients. **F**: TIE-NDMM patients

Matched cohort log-rank analyses showed superior DVd PFS: 12-month PFS 91.9% vs. 84% (HR = 0.36, 95%CI 0.13–0.96, *p* = 0.032, 64% risk reduction; Fig. 3A). DVd produced marked PFS benefits for high-risk cytogenetics (HR = 0.10, *p* < 0.001, 90% risk reduction; Fig. 3B) and ISS III patients (HR = 0.33, 95% CI: 0.11–0.97, *p* = 0.033, 67% risk reduction; Fig. 3C). A non-significant favorable trend existed in patients > 65 years (HR = 0.32, 95% CI: 0.08–1.2, *p* = 0.071, Fig. 3D). DVd significantly prolonged PFS in TE-NDMM patients (HR = 0.21, 95% CI: 0.05–0.97, *p* = 0.028, 79% risk reduction, Fig. 3E), with no difference in TIE-NDMM patients (HR = 0.49, 95% CI: 0.09–2.69, *p* = 0.40). The survival benefits of DVd were consistently observed in high-risk and key populations, highlighting its core value in prolonging long-term survival and delaying disease progression, and compensating for the limited efficacy of VRd in high-risk patients.

#### Mortality events

OS data were immature with few deaths, so formal comparative survival analysis was not performed; only descriptive mortality data are reported. Six deaths occurred overall: four in the DVd arm (severe treatment-related bleeding, post-HSCT complication, progression after maintenance cessation, severe COVID-19) and two in the VRd arm (disease progression, post-HSCT thrombotic microangiopathy with severe infection).

#### Duration of response

**Fig. 4:**
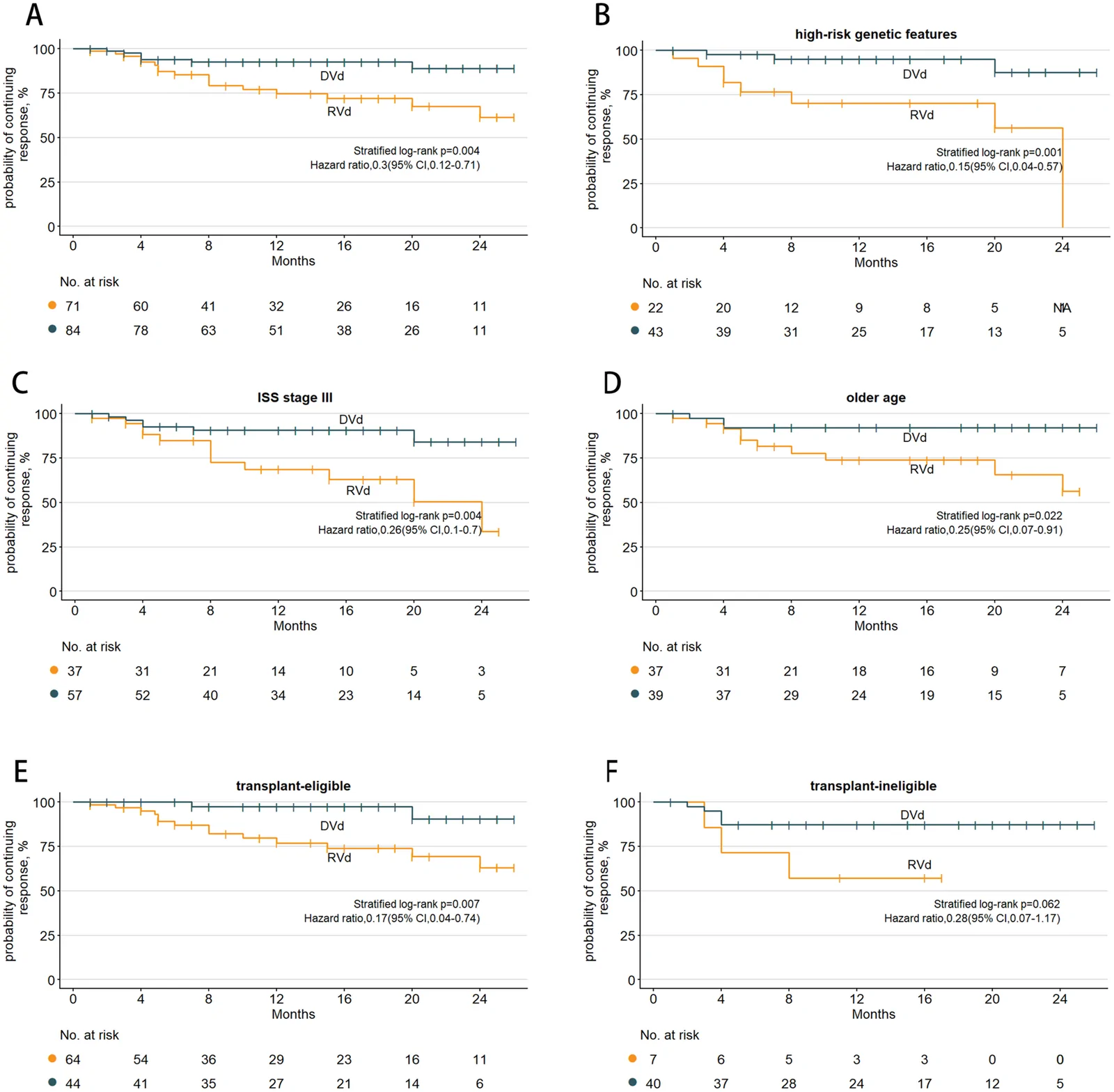
Panels **A**–**D** displayed probability of continuing response comparing the DVd and VRd regimens in the original cohort NDMM patients across distinct subgroups. **A**: The overall cohort of NDMM patients. **B**: NDMM patients with high-risk genetic features. **C**: NDMM patients with ISS stage III disease. **D**: NDMM patients with older age. **E**: TE-NEMM patients. **F**: TIE-NDMM patients

DOR analysis included 155 responding patients excluding transplant recipients (DVd = 84, VRd = 71). Median DOR was unreached for both arms by June 2025. The 12-month DOR rate was significantly higher with DVd (92.5% vs. 74.6%, Fig. 4A). Significant DOR benefits for DVd were consistent across all predefined subgroups (age > 65, ISS III, high cytogenetic risk, transplant eligibility status; Fig. 4B–F), as evidenced by HRs consistently < 1 and statistically significant *p*-values.

**Fig. 5:**
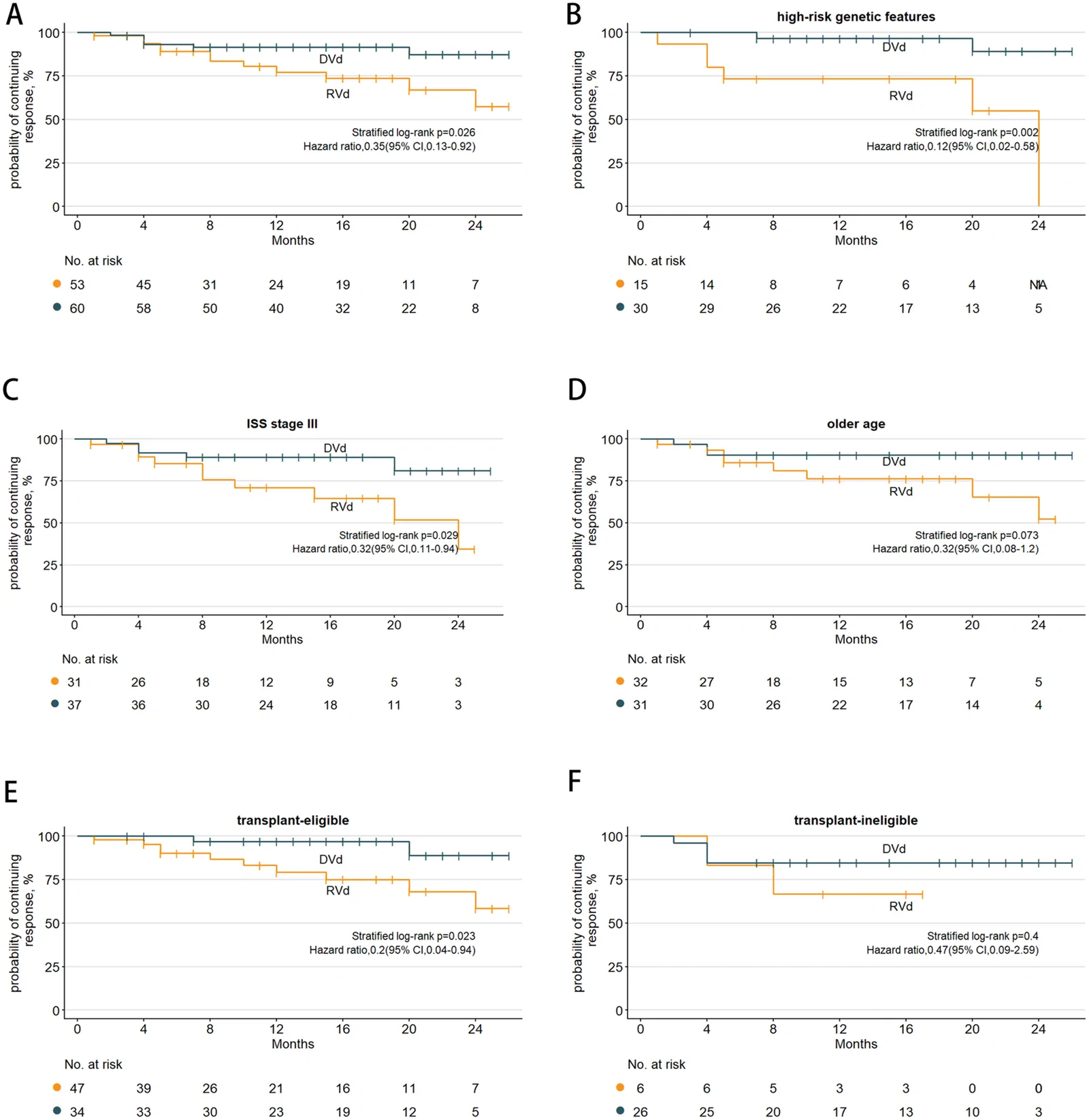
Panels **A**–**D** displayed probability of continuing response comparing the DVd and VRd regimens in the propensity score-matched cohort NDMM patients across distinct subgroups. **A**: The overall cohort of NDMM patients. **B**: NDMM patients with high-risk genetic features. **C**: NDMM patients with ISS stage III disease. **D**: NDMM patients with older age. **E**: TE-NEMM patients. **F**: TIE-NDMM patients. *Limitation: The transplant-eligible and transplant-ineligible cohorts were stratified according to formal HSCT eligibility criteria, and this subgroup analysis was only provided as supplementary information

Log-rank tests confirmed longer DOR with DVd in matched patients: 12-month DOR 91.5% vs. 77.2%, 24-month DOR 87.4% vs. 57.4% (HR = 0.35, 95%CI 0.13–0.92, *p* = 0.026; Fig. 5A). DVd yielded significant DOR improvements for ISS III, high-risk cytogenetics and transplant-eligible patients (Fig. 5B/C/E). Numerically superior DOR without statistical significance was seen in patients aged > 65 years and transplant-ineligible patients (Fig. 5D/F).

### Analysis of factors influencing therapeutic efficacy

Multivariate logistic regression evaluated baseline factors and regimen interactions for response depth. No baseline predictors independently correlated with treatment efficacy: extramedullary lesions (OR 1.196, 95%CI: 0.313–4.575), impaired renal function (eGFR < 60 ml/(min*1.73 m²), OR 1.565, 95%CI: 0.757–3.239), high-risk cytogenetics (OR 1.217, 95%CI: 0.601–2.462). Regimen efficacy remained comparable even in patients with poor baseline prognostic markers.

### Side effects

**Fig. 6:**
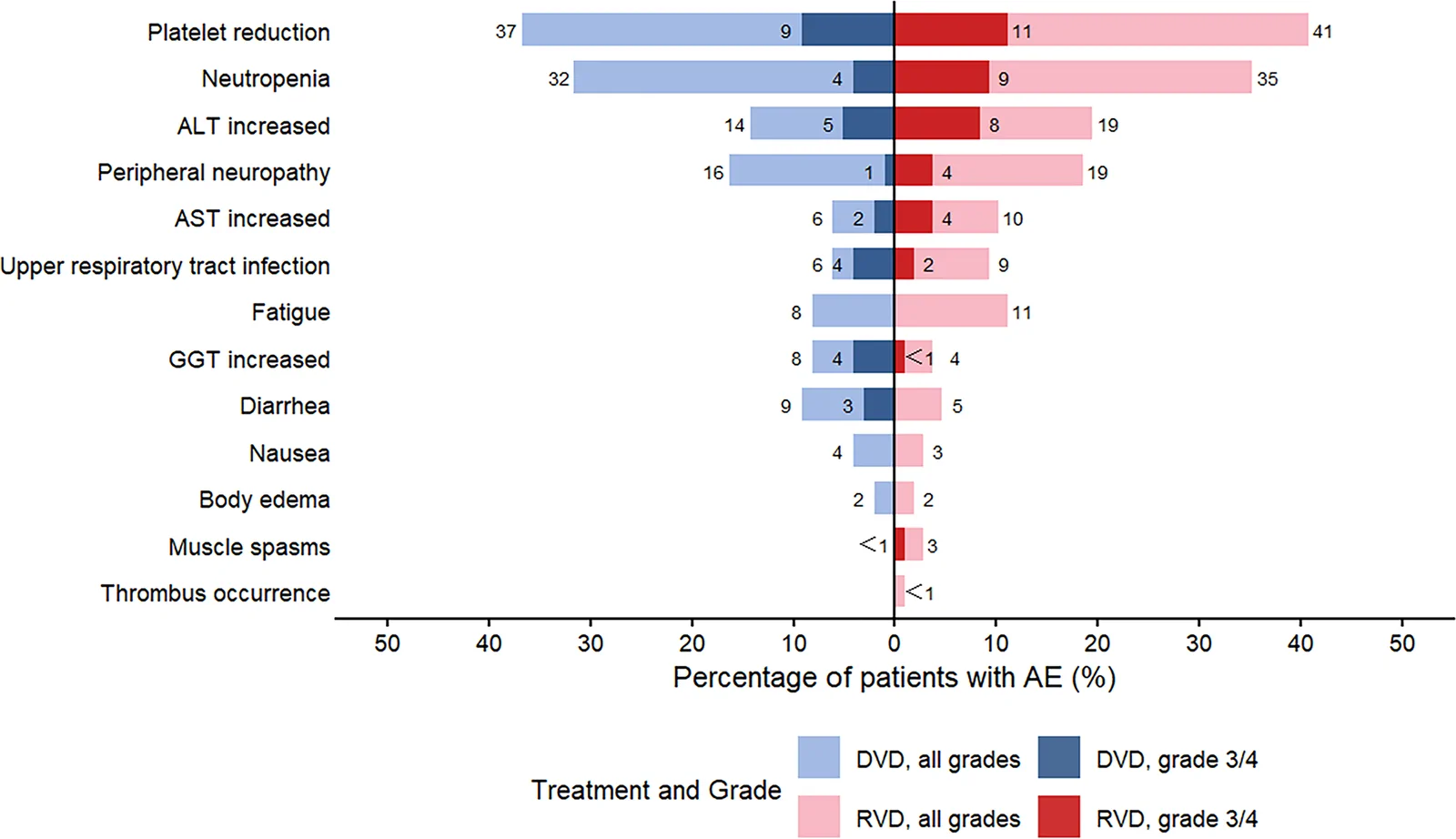
Side effects in the original cohort NDMM patients

AEs were manageable in both arms. Any-grade AEs: DVd 78.57%, VRd 78.70%; ≥grade 3 AEs: 63.27% vs. 64.81%. DVd had slightly more mild neutropenia but less grade 3–4 neutropenia and thrombocytopenia (all *p* > 0.05). In the original cohort, the DVd group had a slightly higher incidence of grade 1–2 neutropenia than the VRd group (27.55% vs. 25.93%, *p* = 0.792). However, the DVd group showed a lower incidence of grade 3–4 neutropenia (4.08% vs. 9.26%, *p* = 0.14). Similarly, for platelet reduction, the DVd group had relatively lower severe (3–4 grade) adverse reaction rates (9.18% vs. 11.11%, *p* = 0.648). For non-hematological adverse reactions, the incidence of grade 1–2 fatigue in the DVd group was lower than that in the VRd group (8.16% vs. 11.11%, *p* = 0.475). Incidence of grade 1–2 upper respiratory tract infection was comparable in both groups (approximately 4%). Severe nonhematologic toxicities (diarrhea, nausea, muscle spasms) occurred less often in DVd. Among grade 1–2 liver function abnormalities, the incidences of elevated ALT, AST, and GGT in the DVd group (4.08% each) were similar to those in the VRd group (ALT: 6.48%, AST: 2.77%, GGT: 2.77%). For 3–4 grade, the DVd group had no AST and GGT elevations and only a 4.08% ALT elevation incidence, while the VRd group had higher rates (ALT: 8.33%, AST: 3.70%, GGT: 3.70%). In terms of thrombus occurrence, the DVd group had a 2.04% incidence of thrombus in 1–2 grade and none in 3–4 grade, whereas the VRd group had a 4.62% incidence in 1–2 grade and 0.92% in 3–4 grade. Peripheral neuropathy and edema rates were comparable (Fig. 6).

**Fig. 7:**
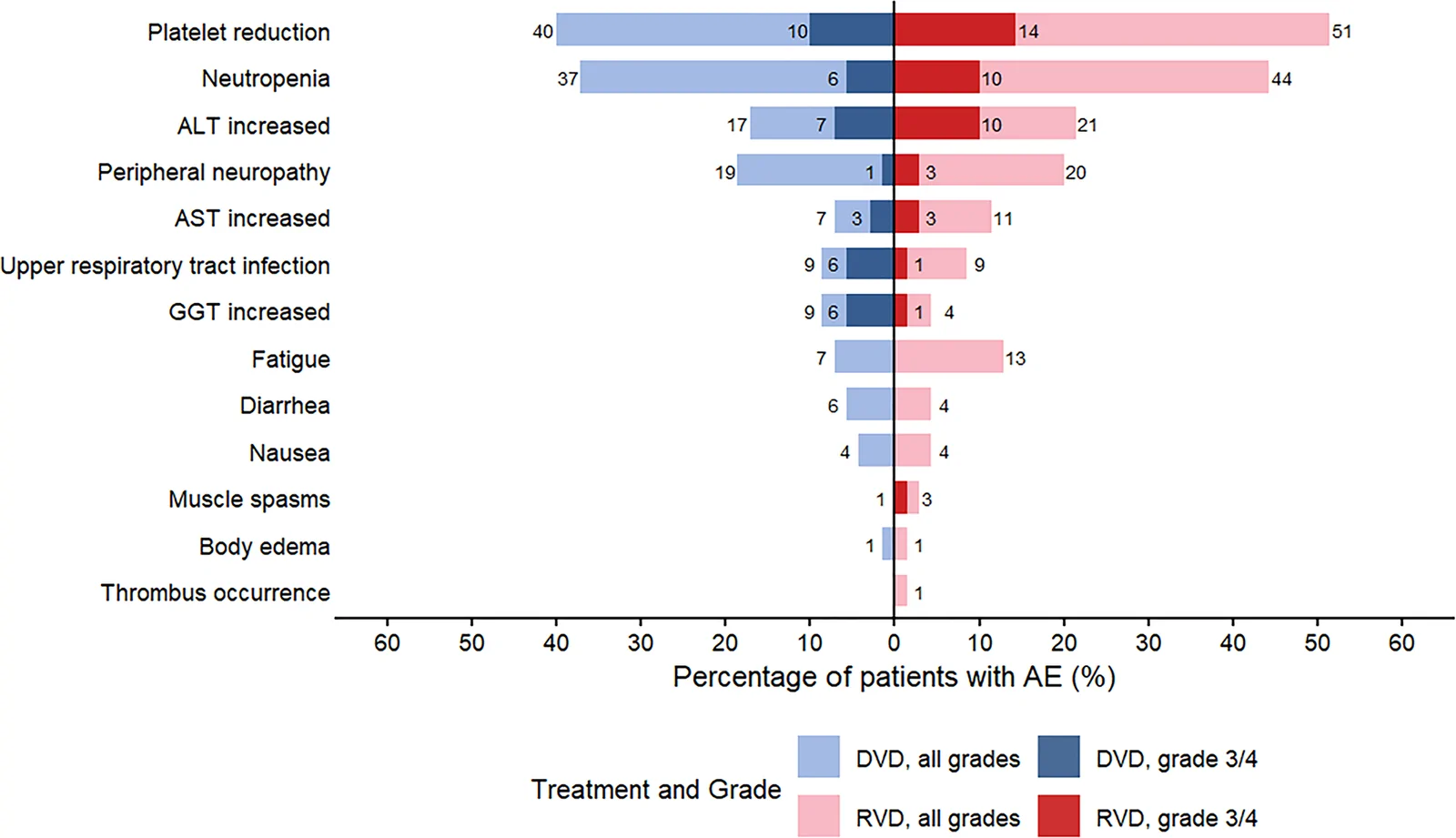
Side effects in the propensity score-matched cohort NDMM patients

In the PSM cohort, DVd exhibited superior hematological tolerability versus VRd. DVd had lower rates of grade 1–2 neutropenia (31.43% vs. 34.29%) and grade 3–4 neutropenia (5.71% vs. 10.00%, *p* = 0.346). It also showed numerically lower grade 1–2 (30.00% vs. 37.14%, *p* = 0.396) and grade 3–4 thrombocytopenia (10.00% vs. 14.29%, *p* = 0.438). For non‑hematological AEs, DVd had milder fatigue (7.14% vs. 12.86%) and no grade 3–4 nausea or muscle spasms, whereas such severe toxicities occurred in the VRd group. For 1–2 grade liver function abnormalities, the incidences of elevated ALT, AST, and GGT in the DVd group (10%, 4.29%, and 3%, respectively) were lower to those in the VRd group (11.43%, 8.57%, and 2.86%). In the 3–4 grade, the two groups had elevation incidences in ALT, AST, and GGT (DVd vs. VRd: 7.14% vs. 10%, 2.86% vs. 2.86%, and 6% vs. 1.43%, respectively). The DVd group had a 1.42% incidence of thrombus in the 1–2 grade and none in the 3–4 grade, compared to the VRd group’s 4.28% in 1–2 grade and 1.42% in 3–4 grade. While mild peripheral neuropathy rates were identical (17.14%) in both groups, DVd had fewer grade 3–4 events (1.42% vs. 2.85%). Edema incidences were similar overall. Collectively, DVd demonstrated milder severe AEs than VRd in both original and PSM cohorts, confirming better overall tolerability for NDMM patients (Fig. 7).

## Discussion

Novel agents have transformed NDMM treatment, yet frontline regimen selection is complicated by heterogeneous prognosis and toxicities. This multicenter prospective study compared DVd and VRd in 206 NDMM patients, using PSM to balance baseline disparities. DVd yielded equivalent short-term remission rates to VRd, but delivered superior long-term disease control with manageable toxicities, supporting its capacity to sustain deep responses. Both regimens achieved four-cycle ORRs over 95%, consistent with established VRd trial ORRs of 82%–90% [24]. As a benchmark triplet, VRd sets a high response threshold that few regimens can exceed. Notably, DVd induced faster remission, rapidly alleviating myeloma-related bone pain, anemia and renal injury while lowering progression risk and optimizing subsequent consolidation. Despite worse baseline risks in the DVd arm (older age, advanced ISS, impaired renal function), deep remission rates (CR/sCR, MRD negativity) matched VRd. Multivariate regression confirmed high-risk features (del(17p), extramedullary lesions) did not attenuate DVd efficacy, indicating DVd may neutralize poor prognostic factors. These results echo the MASTER trial, wherein daratumumab-containing regimens achieved MRD negativity in > 80% high-risk NDMM patients [25, 26]. Daratumumab’s immunomodulatory activity suppresses tumor immunosuppressive microenvironments via PD-L1 downregulation, offsetting the negative impact of high-risk cytogenetics [27]. The 30% ≥CR rate with four-cycle DVd approximated quadruplet DVRd outcomes from GRIFFIN and PERSEUS, yet with a more favorable toxicity burden—an important advantage for frail or elderly patients [28, 29]. DVRd also carries practical drawbacks in Chinese transplant centers: lenalidomide impairs CD34 + stem cell mobilization, delaying adequate cell collection and raising medical costs [30]. Furthermore, quadruplet DVRd requires four concurrent patented agents, generating substantially higher monthly out-of-pocket expenses even after national medical insurance reimbursement, especially for patients without commercial supplementary insurance. However, the rapidly evolving frontline treatment landscape features multiple competing daratumumab-based triplet backbones beyond DVd, including DKd (daratumumab-carfilzomib-dexamethasone) and DPd (daratumumab-pomalidomide-dexamethasone). Although DKd and DPd have shown great efficacy and prolonged survival in indirect comparisons with DVd, these advantages may be counterbalanced by greater toxicity, which is a key consideration in the frontline setting. DKd has cumulative cardiotoxicity and severe thrombocytopenia, restricting its use in patients with baseline cardiac dysfunction, renal impairment, or elderly populations with frailty. DPd relies on pomalidomide rather than bortezomib, delivering robust activity in lenalidomide-refractory disease, yet its weaker upfront response ceiling limits first-line induction as frontline standard, and cumulative myelosuppression increases infectious risk during pre-transplant cytoreduction [31]. 

The most compelling finding from our study is the favorable PFS profile observed in DVd group, with Kaplan-Meier curves demonstrating consistently higher cumulative survival probabilities. This finding aligned with the CASTOR phase 3 trial, where the DVd regimen notably extended PFS in patients with RRMM, thereby establishing a solid foundation for its clinical application. Specifically, in subgroup analysis of TE-NDMM patients, PFS advantage of DVd treatment strongly supported its efficacy as an induction therapy. Efficient induction therapy lays a solid foundation for subsequent stem cell collection, transplantation, and maintenance therapy—an effect that may also be associated with Dara’s immunomodulatory properties [32]. Dara was reported to have the ability in reshaping the tumor microenvironment and induce “immune synergy” by activating immune effector cells such as macrophages and natural killer (NK) cells, thereby achieving deeper disease eradication [33, 34]. This eradication effect showed more prominent advantages in transplant candidates with better physical function. In TIE-NDMM patients, DVd demonstrated a HR < 1, but the difference did not reach statistical significance, potentially due to the small sample size or insufficient number of events during a short follow-up in this subgroup.

DVd also exhibited longer PFS benefits in patients with high-risk cytogenetic abnormalities, ISS stage III disease, and elderly patients. As a CD38 monoclonal antibody, Dara targets myeloma cells through multiple mechanisms, including antibody-dependent cellular cytotoxicity (ADCC), complement-dependent cytotoxicity (CDC), and direct induction of apoptosis. This action synergizes with the pro-apoptotic effects of the proteasome inhibitor bortezomib [35]. Existing literature also indicated that high-risk patients, identified using the SKY92 gene signature combined with FISH detection, showing significant trends toward survival improvement after receiving the DVd regimen [36]. This suggests that Dara may partially overcome adverse genetic prognoses through immune-mediated cellular clearance. In contrast, while the VRd regimen demonstrates efficacy as a standard therapy, the VRd regimen relies on lenalidomide’s cereblon (CRBN)-dependent immunomodulatory activity [37] and bortezomib’s proteasome inhibition. Lenalidomide efficacy is tightly linked to the CRBN pathway, and myeloma cells can develop resistance through CRBN mutations or dysregulation of downstream signaling cascades [38]. This likely contributes to the steeply increasing hazard of progression in the VRd group, as tumor cells escape the therapeutic pressure of lenalidomide more readily. The selection of therapeutic regimens for NDMM patients should be tailored according to the compatibility between pharmacological mechanisms and individual tumor biological characteristics. In patients with high tumor burden and compromised immune function, the multi-target synergistic anti-tumor activity of the DVd regimen confers prominent clinical advantages. This provides a critical translational basis for optimizing individualized therapeutic strategies, whereby well-elucidated mechanisms of action from preclinical research can be translated into core reference criteria for clinical regimen selection.

The DOR analysis from this real-world ITT population of NDMM patients yields compelling evidence of superior durability of DVd compared to VRd. The fact that median DOR remains unreached in both groups (with clear separation in survival curves) suggests DVd confers long-term disease control, a critical unmet need in this incurable malignancy that translates to reduced progression risk, and potentially improved quality of life [39, 40]. This DVd benefit is consistently observed in the PSM cohort across key subgroups: overall population, ISS stage III, high cytogenetic risk, and TE-NDMM patients—reinforcing that the durability advantage of DVd is robust. Crucially, the results supported DVd showed advantages in patients with high-risk features. DOR is strongly correlated with PFS in myeloma and often serves as a surrogate for overall survival (OS), though longer follow-up is needed to confirm OS benefit.

Notably, the PSM cohort reveals a key distinction: no significant difference in DOR between DVd and VRd among elderly patients and TIE-NDMM patients. For these populations, treatment goals often prioritize tolerability and quality of life over maximal response duration; the absence of DOR difference thus supports clinical equipoise, allowing clinicians to select between DVd and VRd based on toxicity profiles, patient preferences, or resource availability rather than durability alone.

Distinct toxicity profiles between the two regimens guide clinical regimen selection. Corresponding to their mechanisms, DVd produced fewer grade 3–4 hematologic toxicities, while VRd carried higher risks of muscle spasms, liver enzyme elevation, thrombosis and peripheral neuropathy—classic adverse effects linked to lenalidomide and bortezomib. No grade 3–4 febrile neutropenia occurred in either arm, and over 94% of patients completed induction, confirming acceptable overall tolerability. DVd’s milder toxicity is driven by daratumumab’s high tumor specificity with limited suppression of normal hematopoietic cells. This safety advantage renders DVd a gentler option for elderly patients, those with multiple comorbidities or compromised bone marrow function. It broadens the population suitable for potent frontline myeloma therapy, balances anti-tumor efficacy and treatment burden, and enables well-tolerated maintenance strategies to sustain long-term survival gains.

Renal impairment is a critical adverse prognostic factor in NDMM. Consistent with lenalidomide’s renoprotective properties [41], VRd yielded numerically better renal responses across all eGFR-stratified renal dysfunction subgroups. However, these intergroup differences failed to reach statistical significance, likely due to small sample sizes, particularly in severe and end-stage renal impairment subgroups. Both regimens had an identical median 1.0-month time to renal recovery; VRd showed only a non-significant trend toward faster mean renal function improvement in both original and PSM cohorts. Comparable eGFR amplification and overall renal response rates confirmed DVd as a viable frontline option for renally impaired NDMM patients. Compared with DVRd, DVd presents superior applicability for patients with high tumor burden and severe renal dysfunction, including dialysis-dependent patients, overcoming DVRd’s limited efficacy in this vulnerable population. Given its non-renal-toxic profile and minimal dose-adjustment requirements, DVd is clinically preferable for myeloma patients with concurrent renal impairment.

This multicenter study improves result generalizability, and propensity score matching offsets baseline confounding in this observational research. Comprehensive cytogenetic detection and MRD evaluation supply detailed data on high-risk disease and deep remission. Divergent PFS results between raw and matched cohorts demonstrate the necessity of statistical adjustment in comparative effectiveness studies Several limitations exist. The non-randomized design inevitably introduces selection bias despite PSM adjustment. Short follow-up prevents definitive long-term survival conclusions; median PFS and DOR remain unreached, and OS data are immature with merely 10 deaths recorded, precluding formal OS comparison and limiting analysis to descriptive mortality data. Still, consistent separation of PFS and DOR curves implies the efficacy superiority of DVd will persist with extended follow-up.

DVd is recommended as preferred first-line therapy for NDMM, especially for patients with renal dysfunction, high-risk cytogenetics and ISS stage III disease. VRd can serve as an alternative for patients with low-risk baseline features to support individualized precise treatment. Subcutaneous daratumumab further boosts the clinical practicality of DVd. Regular monitoring of blood counts and liver function is required for both regimens; close surveillance and timely intervention for severe hematologic toxicities are particularly critical for VRd recipients to balance efficacy and safety. Further randomized controlled trials are needed to verify the efficacy and safety of DVd and VRd. Future long-term follow-up studies will validate OS benefits, conduct cost-effectiveness analyses, and develop MRD-driven individualized treatment algorithms. Translational research integrating basic and clinical science will identify predictive biomarkers, build precision therapy models and realize personalized care. The present findings may also inform updated clinical guidelines to standardize NDMM management and advance overall myeloma treatment strategies.

## Supplementary Information

Below is the link to the electronic supplementary material.

Supplementary Material 1

## Data Availability

The raw de-identified clinical datasets generated and analyzed in this multicenter retrospective propensity score-matched cohort study are not publicly available due to strict institutional patient privacy protection regulations and ethical restrictions. De-identified aggregated analytic datasets supporting the findings of this study are available from the corresponding author upon reasonable written request, following institutional data access approval procedures.
