# Supplementary material for: Superior 12-month progression-free survival with frontline DVd versus VRd in patients with newly diagnosed multiple myeloma: a multicenter propensity score-matched analysis
Source: J Transl Med. 2026 Aug 12;24:1091. doi: 10.1186/s12967-026-08637-6 (PMC13508211; doi:10.1186/s12967-026-08637-6)
Supplement: Supplementary file 1 [file 12967_2026_8637_MOESM1_ESM.docx]

**Study sites**

A total of 12 participating centers across North China were involved in this study, including: the Department of Hematology of Shengjing Hospital of China Medical University (Shenyang, PI: Aijun Liao), Peking University People’s Hospital (Beijing, PI: Jin Lu), Fu Xing Hospital of Capital Medical University (Beijing, PI: Liru Wang), Anshan Central Hospital (Anshan, PI: Qing Leng), Panjin Liaoyou Gemstone Flower Hospital (Panjing, PI: Rong Li), The First Affiliated Hospital of China Medical University (Shenyang, PI: Rui Zhang), Huludao Central Hospital (Huludao, PI: Fei Tian), Panjin Central Hospital (Panjin, PI: Yu Jiang), Liaoning Health Industry Group Bengang General Hospital, (Benxi, PI: Min Wang), Tieling Central Hospital (Tieling, PI: Jingjun Wang), Chaoyang Central Hospital (Chaoyang, PI: Xiaohong Wang), Yingkou Central Hospital (Yingkou, PI: Wenxia Liu).

**Table S1. Detailed renal response categories stratified by baseline eGFR.**

|  |  | **Original cohorts** | | **Pseudo-population** | |
| --- | --- | --- | --- | --- | --- |
| **eGFR subgroup** | **Renal response** | **DVd, N (%)** | **RVd, N (%)** | **DVd, N (%)** | **RVd, N (%)** |
| 30 ≤ eGFR < 60 | renal-CR  reanl-PR  renal-MR  no response | 11 (73.3)  0 (0.0)  0 (0.0)  3 (21.4) | 9 (90.0)  1 (10.0)  0 (0.0)  0 (0.0) | 11 (78.6)  0 (0.0)  0 (0.0)  3 (21.4) | 8 (88.9)  1 (11.1)  0 (0.0)  0 (0.0) |
| 15 ≤ eGFR < 30 | renal-CR  reanl-PR  renal-MR  no response | 4 (26.7)  1 (6.7)  7 (46.7)  3 (20.0) | 5 (71.4)  0 (0.0)  1 (14.3)  1 (14.3) | 2 (22.2)  1 (11.1)  4 (44.4)  2 (22.2) | 5 (71.4)  0 (0.0)  1 (14.3)  1 (14.3) |
| eGFR < 15 | renal-CR  reanl-PR  renal-MR  no response | 5 (21.7)  6 (26.1)  7 (30.4)  5 (21.7) | 1 (25.0)  2 (50.0)  1 (25.0)  0 (0.0) | 1 (20.0)  1 (20.0)  2 (40.0)  1 (20.0) | 1 (25.0)  2 (50.0)  1 (25.0)  0 (0.0) |

**Table S2. Summary of RRR achieved in DVd and RVd cohorts.**

|  | **Original cohorts** | | **Pseudo-population** | |
| --- | --- | --- | --- | --- |
| **Renal response, N (%)** | **DVd （N=53)** | **RVd  (N=21)** | **DVd（N=28)** | **RVd**  **(N=20)** |
| renal-CR | 20 (37.7) | 15 (71.4) | 14 (50.0) | 14 (70.0) |
| reanl-PR | 8 (15.1) | 3 (14.3) | 2 (7.1) | 3 (15.0) |
| renal-MR | 14 (26.4) | 2 (9.5) | 6 (21.4) | 2 (10.0) |
| overall renal response | 42 (79.2) | 20 (95.2) | 22 (78.6) | 19 (95.0) |
| no response | 11 (20.8) | 1 (4.8) | 6 (21.4) | 1 (5.0) |

**Figure S1A: Kaplan-Meier curves for TTRR in the original cohort**

**Figure S1B: Kaplan-Meier curves for TTRR in the propensity score-matched cohort**

**Figure S2: The ethics approval certificate of “new medical technology” study initially approved by the Ethics Committee of Shengjing Hospital of China Medical University in 2023 (Approval No. 2023PS090J)**

| Item | Content |
| --- | --- |
| Ethics No. | 2023PS090J |
| Project Title | Efficacy and Safety Observation of DVd versus RVd Regimens in Patients with Newly Diagnosed Multiple Myeloma |
| Applicant Department | Department of Hematology (Ward 1) |
| Principal Investigator | Jiang Huinan |
| Reviewed Documents | 1. Study Protocol (Version V3, dated 2023.04.19)  2. Feasibility Demonstration Report (Version V1, dated 2023.03.31)  3. Informed Consent Form (Version V3, dated 2023.04.19) |
| Checked Items | ☑ New Medical Technology Protocol  ☑ Informed Consent Form / Waiver of Informed Consent Application  ☑ Operator Qualification Certifications  ☑ Others |
| Review Opinion | For new technologies requiring patient informed consent, implementation shall only be initiated after patients have signed the informed consent forms, and all patient identifiable information shall be de-identified. For new technologies eligible for informed consent waiver, all patient information must be masked to strictly protect patient privacy. |
| Approver (Signed & Ethics Committee Seal) | Zhao Yuhong  Approval Date: 2023-04-21 |
